# Reconstruction of a Chronic Quadriceps Tendon Rupture in an Elderly Polio Patient

**DOI:** 10.3390/biomedicines13061363

**Published:** 2025-06-01

**Authors:** Mario Ronga, Leonardo Callegari, Francesco Roberto Evola, Camilla Crespi, Lia Rimondini, Davide Ciclamini

**Affiliations:** 1 Orthopaedic and Trauma Operative Unit, Department of Health Sciences, University of Eastern Piedmont, Via Paolo Solaroli, 17, 28100 Novara, Italy; davide.ciclamini@uniupo.it; 2 Department of Radiology, ASST Sette Laghi, Ospedale di Circolo, Viale L. Borri 57, 21100 Varese, Italy; leonardo.callegari@asst-settelaghi.it; 3 Department of Orthopaedic and Trauma Surgery, Cannizzaro Hospital, University of Catania, 95100 Catania, Italy; robertoevola@virgilio.it; 4 Center for Translational Research on Autoimmune and Allergic Diseases, Department of Health Sciences, University of Eastern Piedmont, Via Paolo Solaroli, 17, 28100 Novara, Italy; lia.rimondini@med.uniupo.it

**Keywords:** quadriceps tendon, chronic tendon tear, poliomyelitis, knee

## Abstract

Quadriceps tendon tears are uncommon lesions and their diagnosis can be missed in up to 50% of cases. We report a case of a 83-year-old man with polio who has a chronic quadriceps tendon tear in a healthy limb. A modified Codivilla technique augmented with an ipsilateral semitendinosus tendon was performed. Partial weight bearing was allowed immediately after surgery, and at 3 months the patient was able to walk with full weight bearing on the involved limb. The 5-year follow-up MRI showed good quadriceps tendon healing with the full integration of the tendon graft. Tendon repair with autograft augmentation represents a potential and effective solution in elderly patients with a chronic quadriceps tendon tear.

## 1. Introduction

Chronic quadriceps tendon tears are uncommon lesions and their real incidence is not reported in the literature [1,2,3]. The diagnosis of quadriceps tendon rupture may initially be misdiagnosed with incomplete rupture, unstable patella, and neurological conditions in between 10% and 50% of cases [4,5]. The treatment of these is challenging due to several factors such as defect size, muscle retraction, muscle adhesion to the deep layers, tendon and muscle myxoid degeneration and the poor quality of repair tissue [1,2,6]. There is no consensus on which is the best method in chronic injury management, and to the best of our knowledge, no cases have been reported in the literature on chronic quadriceps tendon lesions in polio patients. In the context of polio, “unilateral sufficiency” refers to a state where only one side of the body or a specific limb is affected by the disease and the recovery of eventual lesions of the unaffected side is fundamental for the quality of life [7]. We report a case of an elderly polio patient with a chronic quadriceps tendon rupture of an unaffected limb. This clinical scenario could lead to significant functional impairments and a reduction in quality of life without proper treatment. Our original surgical technique and the results at the medium-term follow-up are reported.

## 2. Case Report

**Figure 1:**
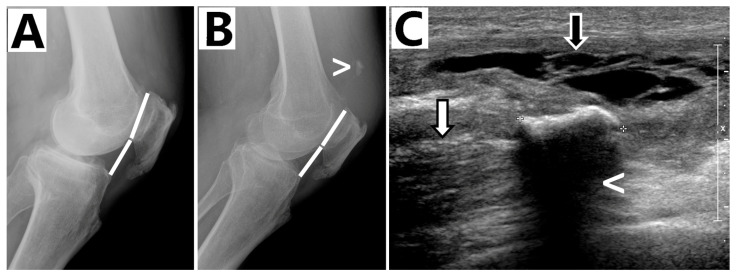
(**A**) **Caton–Deschamps Index values** at trauma of 0.8; (**B**) **Caton–Deschamps Index values** at pre-op of 0.73. Enthesophyte of the quadriceps tendon (>); (**C**) **Ultrasound scan (Pre-operative):** Partially organized hematoma with some echogenic septa inside (black arrow); entesophyte (<) of the quadriceps tendon avulsed from the upper pole of the patella and retracted proximally (white arrow).

An 83-year-old man affected by poliomyelitis of the right limb reported onset pain and an inability for active extension of the healthy left knee after a hyperflexion knee movement during gardening. He was under hypertension triple therapy and no drugs predisposing tendon injuries, such as fluoroquinolones, cortisone injections, etc., were used in the past. He was admitted to another receiving hospital and immobilized in a cast for 6 weeks after physical examination and X-ray (Figure 1A). During the rehabilitation phase, he complained of pain and an inability to walk; his general practitioner referred him to our hospital 10 weeks after the trauma. During the physical examination, a palpable suprapatellar gap was detected and an active knee extension lag with a passive range of motion (ROM) of 0–45° was recorded. The Lysholm score was 30/100. The Caton–Deschamps Index value was 0.73 (Figure 1B) and the ultrasound scan showed an insertional full thickness tear of the quadriceps tendon with a proximal retraction of 7 cm (Figure 1C).

## 3. Surgical Technique

The surgery was performed under general anesthesia with the patient in a supine position. Cefazolin 2 gr. iv pre-operatively was selected as a antibiotic prophylaxis. A pneumatic tourniquet was placed on the proximal thigh with the hip and knee flexed.

**Figure 2:**
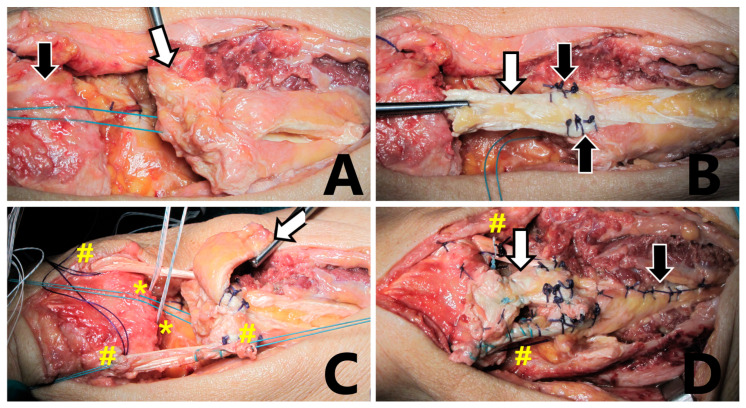
**Surgical procedure:** (**A**) The quadriceps tendon (white arrow) is pulled down after scar tissue debridement and suprapatellar pouch release. The residual gap measured approximately 4 cm. The patella is indicated by the black arrow. (**B**) A modified Codivilla technique: the central rectangular flap (white arrow) is sculpted and overturned, with stitches securing the end part of the plasty (black arrows). (**C**) A suture anchor is in place (*). A semitendinosus graft is passed through the patella tunnel and the quadriceps tendon (#). Codivilla rectangular flap is indicated by the white arrow. (**D**) Final view: codivilla rectangular flap (white arrow); semitendinosus graft (#); side-to-side suture of the proximal quadriceps tendon (black arrow).

A straight longitudinal incision of 15 cm was made over the quadriceps tendon. After sharp dissection, abundant scar tissue was observed at the tear level. The scar tissue debridement was performed and a full thickness defect of 8 cm in length with proximal retraction of the muscle was measured. After suprapatellar pouch release, the quadriceps was mobilized using a #2 Ethibond suture (Ethibond Excel^®^ polyester suture, Johnson & Johnson Ethicon, Cincinnati, OH, USA). The residual tendon gap measured about 4 cm in length (Figure 2A). A direct tendon repair was not possible to perform. The quadriceps tendon reconstruction was performed using a modified Codivilla technique augmented with an ipsilateral semitendinosus graft. A central full thickness rectangular flap (7 × 1.5 cm) of the quadriceps tendon was sculpted, properly stitched and overturned (Figure 2B). A transverse trough was made in the superior pole of the patella with a bur. A transverse incision was made over the pes anserinus and the semitendinosus tendon was harvested with a tendon stripper.

A 6 mm transverse patellar tunnel was drilled and the passage of the free graft was achieved. The distal tendon free part was passed in a transverse manner through the proximal part of the quadriceps tendon and then was tensioned. The central part of the overturned quadriceps was then reattached on the proximal pole of the patella using 2 anchors (Ø5 mm, Super Revo, Linvatec, Largo, FL, USA) with the knee in full extension (Figure 2C). The terminal ends of the semitendinosus were sutured to each other using a non-absorbable suture (#2 Ethibond). A #2 PDS^®^ II absorbable suture (polydioxanone suture, Johnson & Johnson Ethicon, USA) was folded twice and passed in the same manner of the semitendinosus to strengthen the reconstruction temporarily. The proximal quadriceps tendon defect was repaired by performing a side-to-side suture (#1 VICRYL^®^—polyglactin suture, Johnson & Johnson Ethicon, USA) as described by Codivilla in his original technique. The repair was tested at 30° of knee flexion and gapping did not occur (Figure 2D).

**Figure 3:**
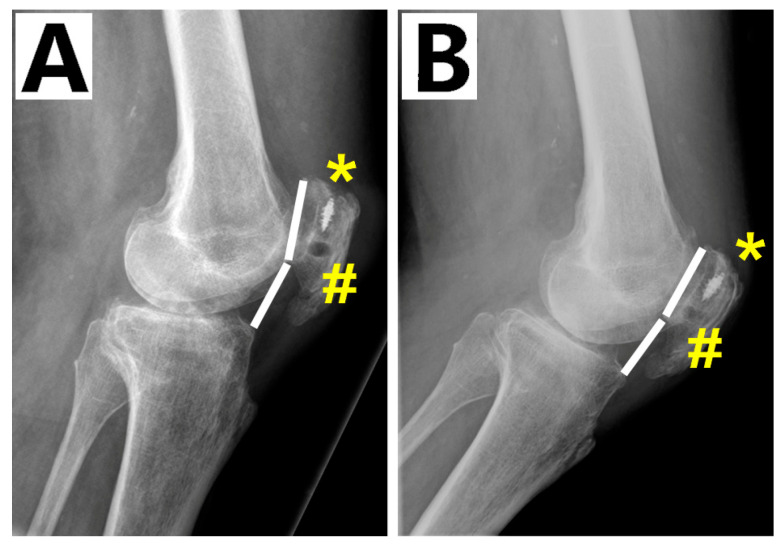
(**A**) Caton–Deschamps Index values: (**A**) 1.07 at post-op; (**B**) 1.04 at 5-year follow-up. suture anchor (*); patella tunnel (#).

The wound was closed in layers and routine dressings, bandages, and a straight knee splint was applied. A drainage in suction was used for 2 days. The post-operative Caton–Deschamps Index was 1.07 (Figure 3A).

In the post-operative period, the knee was immobilized in a hinged brace locked in extension for 1 month, isometric strengthening started immediately, and partial weight bearing was allowed using a walker. After 1 month, the patient was allowed to unlock the brace for active recovery of the knee range of motion and started progressive weight bearing using crutches. At 2 months of follow-up, the knee brace was removed, the patient reached full weight bearing using a walking stick and active knee movement against gravity. At 3 months, the patient had an active knee ROM of −5° in extension and 95° in flexion with movements against gravity and resistance (Medical Research Council—MRC—grading 4/5) [8]. The Lysholm score was 77/100.

**Figure 4:**
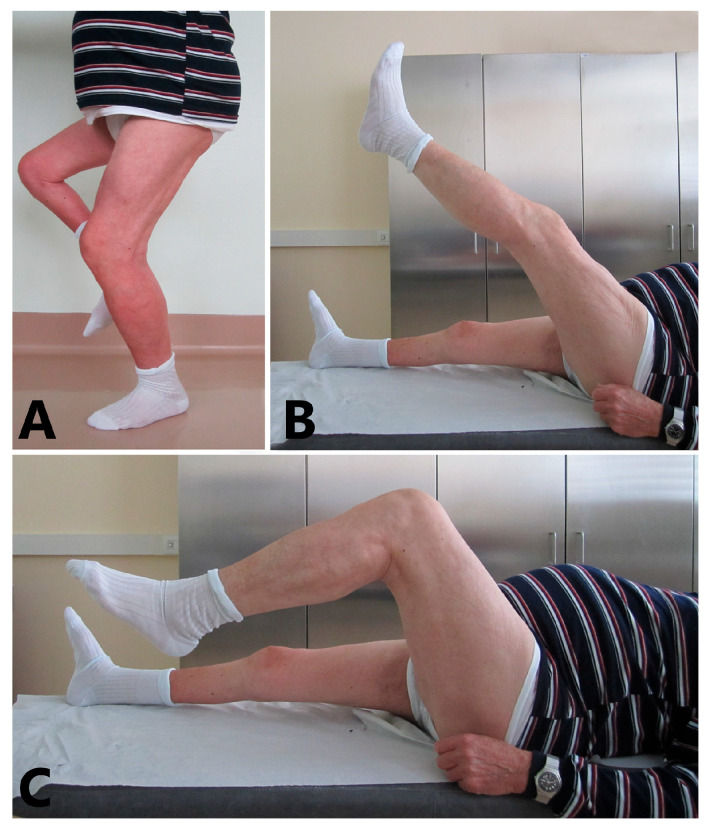
**Follow-up visit at 5 years:** (**A**) The patient is performing a single-leg dip on the treated leg. (**B**) Patient shows −5° of knee extension against gravity. (**C**) Patient shows 95° of knee flexion.

**Figure 5:**
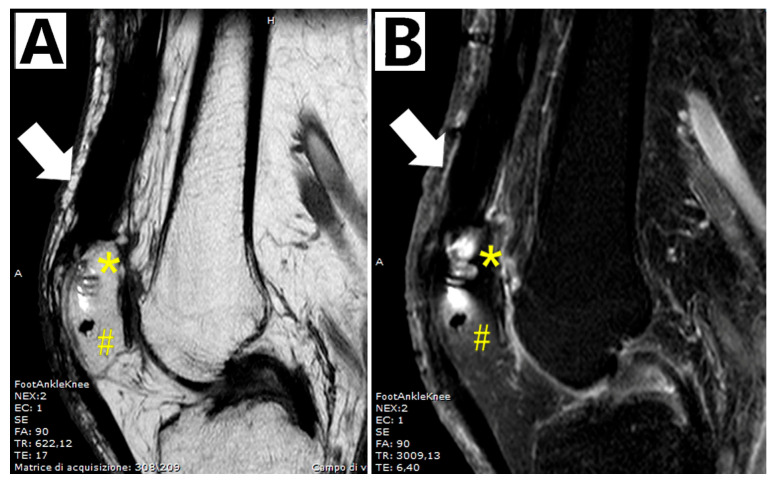
**MRI at 5-year follow-up.** (**A**) Spin-echo (SE), T1-weighted; (**B**) fast spin-echo, fat-saturated, T2-weighted. Continuity of the reconstructed quadriceps tendon with complete graft incorporation: quadriceps tendon (white arrow); suture anchor (*); patella tunnel (#).

At 2 and 5 years follow-ups, the active knee ROM, movements against gravity and resistance and Lysholm score were comparable to those of the previous follow-up (Figure 4) and the MRI showed quadriceps and graft healing (Figure 5). The Caton–Deschamps Index at 5 years was 1.04 (Figure 3B).

## 4. Discussion

Several factors had to be considered to address this particular case: the chronic pattern of the tear, the poor healing process of an elderly patient, and the unaffected limb involvement of a polio patient with consequential possible complication during the rehabilitation period. Due to the uncommon nature of these lesions, the literature lacks a description of the ideal surgical management [1,2,4]. To the best of our knowledge, no similar case has been reported in the literature.

After scar tissue debridement, the residual gap was managed using a modified Codivilla technique. We created the flap in a rectangular shape instead of a triangular one, as described in the original technique, to increase the contact area between the tendon and the proximal patellar pole. The residual proximal quadriceps tendon defect was sutured without any difficulties as described in the original Codivilla Technique [9].

The reconstruction was strengthened with a tendon autograft because of the poor quality of the quadriceps tendon and to reduce the risk of tendon re-rupture. The orientation of the two arms of the autograft reproduced the medial and the lateral portions of the quadriceps tendon at the junction with the extensor retinaculum. This is essential to decrease the failure rate and maximize outcomes [1,2,10]. An allograft was not considered due to its lower biomechanical properties, longer time of integration and poorer biological response in elderly patients compared to an autograft [3,11].

Leopardi et al. [12] reported a technique using the gracilis and the semitendinosus tendon through a patellar tunnel. The free distal parts of the tendons were crossed through the quadriceps tendon several times. At the 37-month follow-up, a lack of 5° in knee active extension and a patella baja were observed. We theorize that the patella baja and the inadequate knee recovery could be due to the low strength of the reconstruction and the lack of continuity between the quadriceps tendon and the superior pole of the patella that are achievable with a Codivilla technique.

Druskin et al. [13] described the use of a patella–quadriceps tendon allograft. Recombinant human bone morphogenetic protein-2 was used at the patellar fixation site in order to improve bone healing. At 13 months, the authors observed a 20° extension knee lag. We theorized that the inadequate knee recovery is due to a lower biomechanical property and longer time of integration of the allograft compared to the autograft [9]. Moreover, the high cost of the bone morphogenetic protein must be considered.

McCormick et al. [14] described a technique using three hamstring autografts harvested from both legs. The free tendons were passed into the quadriceps muscle, folded and then introduced in three separate patellar half tunnels. The fixation was performed through transosseous sutures at the inferior pole of the patella. At the 1-year follow up, the patient had 0°/110° of ROM, no extension lag and could perform weight bearing without any device. However, tendons harvested from a polio limb have low strength and three half tunnels could cause a patella fracture. For these reasons, we used only one hamstring tendon autograft passed through one patellar tunnel to avoid the above-mentioned potential complications.

Rehman et al. [6] reported the results at the short-term follow-up of a revision technique using V-shaped Codivilla plasty augmented by three hamstring autografts, prolene mesh and platelet-rich plasma (PRP). The aim of this procedure was to achieve a stable reconstruction and to improve the graft integration. At the 1-year follow-up, the patient had −10°/90° of ROM, and could perform weight bearing with a single stick. Despite the clinical results being similar to our case, the donor site morbidity of both legs, potential foreign body reaction due to synthetic mesh and the cost of a PRP procedure must be considered.

Sartorius muscle transfer has recently been used in two different case series to treat chronic quadriceps tendon rupture [15,16]. Although good short- and medium-term results were achieved, the rate and type of complications must be taken into account. Elhessy et al. [15] reported complications in all five patients treated, ranging from wound necrosis to patellar necrosis requiring new surgeries.

The success of our technique (modified Codivilla plasty augmented with a biological cerclage) was demonstrated by the functional and imaging results reported in the unaffected limb of this elderly polio patient. The strength of our reconstruction was proven by the good knee recovery, the early weight bearing on the treated limb, the restoration of the normal patella height and the healing and the integration of the biological cerclage. We suggest this procedure for patients with high functional demands affected by a chronic quadriceps tendon tear. However, only prospective comparative studies could demonstrate the superiority of this technique over the others.

## Data Availability

The original contributions presented in this study are included in the article. Further inquiries can be directed to the corresponding author.
